# Association of air pollution with postmenopausal breast cancer risk in UK Biobank

**DOI:** 10.1186/s13058-023-01681-w

**Published:** 2023-07-13

**Authors:** Carmen Smotherman, Brian Sprague, Susmita Datta, Dejana Braithwaite, Huaizhen Qin, Lusine Yaghjyan

**Affiliations:** 1 Department of Epidemiology, College of Public Health and Health Professions and College of Medicine, University of Florida, 2004 Mowry Road, Gainesville, FL 32610 USA; 2 Department of Surgery, University of Vermont, Burlington, VT USA; 3 Department of Biostatistics, College of Public Health and Health Professions and College of Medicine, University of Florida, Gainesville, FL USA

**Keywords:** Air pollution, Breast cancer, UK Biobank, Prospective cohort

## Abstract

**Background:**

We investigated the association of several air pollution measures with postmenopausal breast cancer (BCa) risk.

**Methods:**

This study included 155,235 postmenopausal women (of which 6146 with BCa) from UK Biobank. Cancer diagnoses were ascertained through the linkage to the UK National Health Service Central Registers. Annual exposure averages were available from 2005, 2006, 2007, and 2010 for NO_2_, from 2007 and 2010 for PM_10_, and from 2010 for PM_2.5_, NO_X_, PM_2.5–10_ and PM_2.5_ absorbance. Information on BCa risk factors was collected at baseline. Cox proportional hazards regression was used to evaluate the associations of year-specific and cumulative average exposures with BCa risk, overall and with 2-year exposure lag, while adjusting for BCa risk factors.

**Results:**

PM_10_ in 2007 and cumulative average PM_10_ were positively associated with BCa risk (2007 PM_10_: Hazard ratio [HR] per 10 µg/m^3^ = 1.18, 95% CI 1.08, 1.29; cumulative average PM_10_: HR per 10 µg/m^3^ = 1.99, 95% CI 1.75, 2.27). Compared to women with low exposure, women with higher 2007 PM_10_ and cumulative average PM_10_ had greater BCa risk (4th vs. 1st quartile HR = 1.15, 95% CI 1.07, 1.24, p-trend = 0.001 and HR = 1.35, 95% CI 1.25, 1.44, p-trend < 0.0001, respectively). No significant associations were found for any other exposure measures. In the analysis with 2-year exposure lag, both 2007 PM 10 and cumulative average PM10 were positively associated with BCa risk (4th vs. 1st quartile HR = 1.19, 95% CI 1.10, 1.28 and HR = 1.29, 95% CI 1.19, 1.39, respectively).

**Conclusion:**

Our findings suggest a positive association of 2007 PM_10_ and cumulative average PM_10_ with postmenopausal BCa risk.

**Supplementary Information:**

The online version contains supplementary material available at 10.1186/s13058-023-01681-w.

## Introduction

Established breast cancer risk factors explain only 30–50% of breast cancer cases [1–5], and previous studies in immigrants have demonstrated the importance of environmental factors in the etiology of breast cancer [6]. Air pollution is classified as a human carcinogen by the International Agency for Research on Cancer (IARC) with strongest evidence for lung and bladder cancers, and some studies linking it also to the risk of liver, gastric, cervical, and brain cancers [7–13]. However, the relationship between air pollution and breast cancer remains unclear. Notably, more than half of the worlds’ population continue to be exposed to increased levels of air pollution [14].

Of the common air pollutants used for air quality monitoring, particulate matter (PM), and nitrogen oxides (NOx), including nitrogen dioxide (NO_2_) are of interest with respect to breast carcinogenesis. PM, including fine inhalable particles of ≤ 2.5 µm in diameter (PM_2.5_) and inhalable particles ≤ 10 µm in diameter (PM_10_), have biological properties relevant to breast carcinogenesis and other chronic diseases [15–22], while NOx and NO_2_ represent biomarkers of exposure to PM, polycyclic aromatic hydrocarbons (PAHs) or benzene from traffic related air pollution [23]. Despite known carcinogenic properties of PM constituents, experimental evidence and strong biological plausibility, the epidemiologic evidence on the association of PM with breast cancer remains very limited and inconsistent. Some previous studies reported positive associations of PM_10_ and PM_2.5_, while others found none [24–34]. However, most of the previous observational studies were relatively small (< 3450 breast cancer cases) and/or limited to specific sub-populations of women (nurses or breast cancer-free women with a family history of breast cancer) [9, 24–28, 35–38]. Similarly, for NO_2_, some studies found significant associations, while others found no associations with breast cancer, often with trends suggesting a positive association [26, 27, 31–33, 35–38].

Epidemiologic evidence for biological role of the individual chemicals found in PMs suggests an increase in breast cancer risk in different populations, some even at the lowest detectable levels [39–48]. PAHs have endocrine disrupting properties and interfere with normal DNA damage repair [15–18, 49, 50]. Among other compounds found in PMs, polychlorinated dibenzodioxins (dioxin), dibenzofurans (PCDF), polychlorinated biphenyls (PCB) and heavy metals (cadmium, arsenic, and mercury) have also been shown to have endocrine disrupting properties and associations with breast cancer in some studies [16, 51–59].

Although their intrinsic carcinogenicity is not clearly established, NOx and NO_2_ represents biomarkers of exposure to diesel exhaust, which contains many carcinogenic components such as PM, PAHs, and benzene [60, 61]. An important source of NOx and NO_2_ is fossil fuel combustion, mainly from engine vehicles and energy production; therefore, NOx and NO_2_ are considered as the best road traffic tracers and markers of exposure to components with plausible biological mechanisms, without being directly involved in cancer pathophysiology. Further, it has been previously shown that air concentrations of nickel or vanadium in PM_2.5_ were more highly correlated with NO_2_ or NOx concentration levels than with total PM_2.5_ concentration levels [62]. Thus, NO_2_ might be a better marker of exposure to specific PM species or heavy metals with carcinogenic properties found in vehicles exhaust than ambient total PM concentration levels.

The goal of this study was to explore the association between air pollution (PM_2.5_, PM_10_, PM_coarse 2.5–10_, PM_2.5 absorbance_, NO_2_, and NOx) and breast cancer risk in a large population-based sample of postmenopausal women from an established prospective cohort. These associations were examined overall as well as with 2-year and 5-year exposure lag.

## Methods

### Study population

Women in this study were selected from UK Biobank, an established population-based prospective cohort. UK Biobank contains more than 500,000 (44% women) volunteers who were aged 40–69 years when recruited during 2006–2010 from England, Scotland and Wales via National Health Service (NHS) patient registers [63]. A detailed description of the enrollment process has been previously described [64]. Briefly, at enrollment all participants provided health, lifestyle, and socio-demographic data through questionnaires and interviews, underwent physical examination, provided blood, urine and saliva samples and agreed to be followed for health outcomes. Between 2012 and 2013, 20,346 (20%) participants completed their first repeat assessment. Participants’ outcomes were ascertained via record linkage to the NHS Central Registers. The latest cancer registry record linkage to UK Biobank data was completed on February 29, 2020 for participants from England and Wales, and on August 31, 2021 for Scotland.

As premenopausal and postmenopausal breast cancers are different and as the follow-up data did not capture updates on woman’s menopausal status or reproductive history, only postmenopausal women were included in this study. Women were considered to be postmenopausal at baseline if they reported (1) having menopause (periods stopped), (2) bilateral oophorectomy, or (3) hysterectomy with one or both ovaries retained and being 54 years or older for ever smokers or 56 years or older for never smokers [65]. We included postmenopausal women without a history of breast cancer or any other type of cancer (except non-melanoma skin) at recruitment. A breast cancer diagnosis (invasive and in-situ) was identified based on diagnostic codes according to the 9th revision of the International Classification of Diseases (ICD-9: 174, 2330) or the 10th revision (ICD-10: C50, D05).

**Fig. 1:**
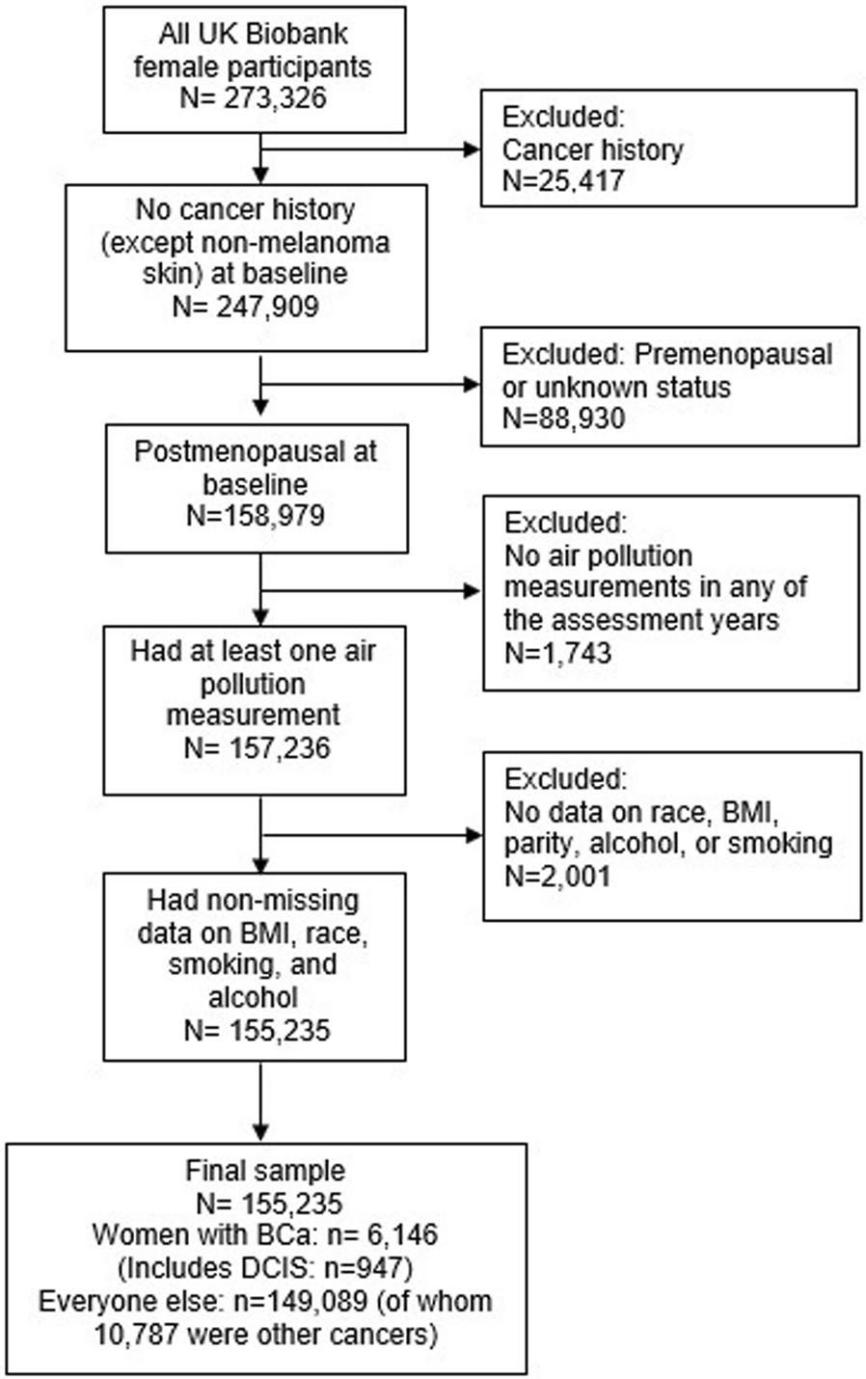
Study sample selection diagram

Out of 158,979 eligible women, we further excluded participants with missing information on all air pollution assessments and selected breast cancer risk factors. The final sample included 155,235 women (98% of all eligible women in UK Biobank) of which 6,146 developed breast cancer during the follow-up through the last linkage to the cancer registries (Fig. 1). UK Biobank protocol was approved by the NorthWest Multi-centre Research Ethics Committee (MREC), which covers the UK. All participants of UK Biobank provided written consent at recruitment.

### Air pollution data

**Fig. 2:**
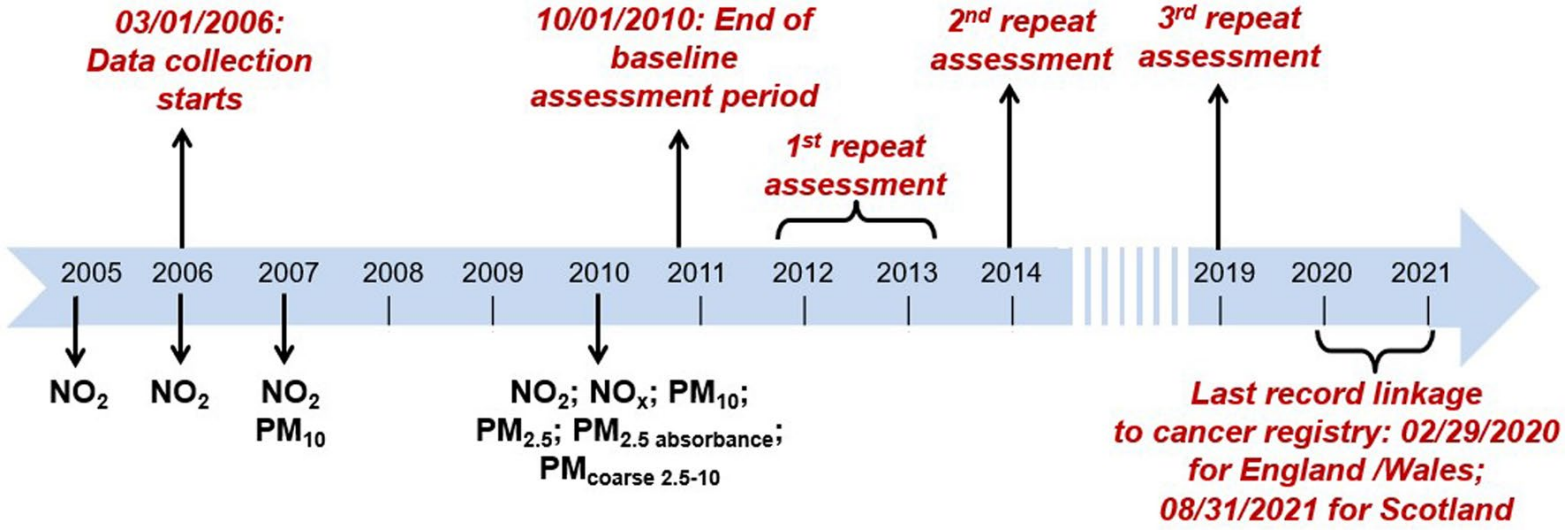
Exposure data collection

In the UK Biobank, the annual averages of PM_10_ and NO_2_ were available for the baseline assessment period (2005, 2006, 2007, and 2010 for NO_2_; 2007 and 2010 for PM_10_), while PM_2.5_, NO_X_, PM coarse (PM with an aerodynamic diameter > 2.5 µm but ≤ 10 µm, PM_2.5–10_) and PM_2.5_ absorbance (measurement of the blackness of PM_2.5_ filters, a proxy for elemental carbon [the dominant light absorbing substance]) were available only for 2010 (Fig. 2).

Air pollution estimates for 2005–2007 were derived from EU-wide air pollution maps (resolution 100 m × 100 m) [66]. The X and Y coordinates of UK Biobank participants were overlaid with these maps (projected to British National Grid) and the corresponding air pollution concentration of the 100 m × 100 m grid cell was assigned to the coordinate. EU-wide air pollution maps were modelled based on a land use regression (LUR) models for Western Europe [66]. The dependent variables in LUR models were ambient concentrations of NO_2_ and PM_10_, obtained from EuroAirnet, the regulatory air pollution monitoring network in Europe [67]. Air pollution estimates for 2010 were modeled for each address using a LUR models developed as part of the European Study of Cohorts for Air Pollution Effects (ESCAPE) [68, 69]. Within the ESCAPE project, PM_2.5_, PM_10_, PM_2.5_ absorbance, and nitrogen oxides (NO_2_ and NOx) were measured between October 2008 and April 2011 during standardized specific PM monitoring campaigns [70, 71]. The estimates from the LUR models were used to calculate the annual averages of air pollutants. The LUR estimates for PM were not valid beyond 400 km from Greater London, the initial ESCAPE study area; therefore, participants living beyond 400 km (mainly from northern England or Scotland), were not assigned PM_10_, PM_2.5_, PM_2.5_ absorbance, and PM_coarse_ concentrations for 2010 to prevent exposure misclassification.

### Covariates information

Information on breast cancer risk factors was extracted for all the participants from baseline questionnaires, interviews, and physical examinations (2006–2010). The following covariates were included: race/ethnicity, Body Mass Index (BMI), age at menarche, age at menopause, parity/age at first child’s birth, postmenopausal hormone therapy, alcohol use, smoking status, and family history of breast cancer. Since there was a small proportion (< 0.5%) of women with missing values on race, BMI, parity, alcohol, and smoking, those women were excluded from the sample, while for variables with relatively larger proportion of missing values (> 3%), we created an “Unknown” category to retain these observations in the models.

For variables that could potentially change with time in postmenopausal women (BMI, alcohol use, smoking, and family history of breast cancer), we found a high correlation in the values for these covariates at baseline with those collected at the repeat assessments on the subset of participants thus justifying the use of baseline data in the analysis. The correlations between baseline values and values collected at first, second, or third subsequent assessments were 0.92, 0.89, and 0.86 for BMI, 0.91, 0.88, and 0.89 for smoking status, 0.76, 0.70, and 0.69 for alcohol use status, respectively; for family history (yes/no), the agreement between first and second assessment was 0.94.

### Statistical analyses

Cox proportional hazards regression models were used to analyze the association between air pollution and breast cancer risk while adjusting for known breast cancer risk factors: age at recruitment (years, continuous), age at menarche (years, continuous), BMI (kg/m^2^, continuous), race (Caucasian [reference], other), parity/age at first child’s birth (nulliparous [reference], parous with age at first birth ≤ 25 years, parous with age at first birth > 25 years, parous with unknown age at first birth), family history of breast cancer in first degree relatives (none [reference], any, unknown), age at menopause (< 46 [reference], 46 to < 50, 50 to < 55, ≥ 55 years, unknown), postmenopausal hormone use (never [reference], past, current, unknown), smoking (never [reference], past, current), and alcohol consumption (never [reference], past, current). The risk estimates were presented as hazard ratios (HRs) and their corresponding 95% confidence intervals (CIs). The regression models were developed for all available measures of air pollution preceding the breast cancer diagnosis in two ways. First, air pollution from specific years was examined in relation to breast cancer risk. Second, the cumulative average exposure for pollutants with multiple assessment years (PM_10_ and NO_2_) was used in the analyses, calculated as the average across all available measures. Each analysis included only incident breast cancer cases diagnosed in the same year or after the measurements of the air pollutant modeled were taken. The follow-up start time and the subset of women included in each analysis listed in Additional file 1: Table 1 are based upon the measure of air pollutant modeled. For example, when analyzing the associations of NO_2_ 2006 with breast cancer risk, the follow-up started in 2006 and included all women who were cancer-free as of 2006, while analysis of NO_2_ 2010 had a start of follow-up in 2010 and included only women who were cancer-free as of 2010. For analyses with the cumulative average exposure to PM_10_ or NO_2_, the follow-up time started at the last available exposure assessment year. In all the analyses, the follow-up time ended at the time of breast cancer diagnosis for women with breast cancer, at the time of death for women who died during the follow-up, at the time of cancer diagnosis for women who developed other cancer type during the follow-up, or at time of the last linkage to the cancer registries for everyone else.

Air pollution was modeled as a continuous variable and the estimates were reported per 5 µg/m^3^ and 10 µg/m^3^ increase of air pollutant, consistent with previous studies [24–29, 32, 33, 37]. Air pollution was also modeled as quartiles based on the distribution in the study sample, specific to assessment year or cumulative average exposure for PM_10_ and NO_2_. Tests for trends were performed using the median level of the exposure within each of the quartiles. Additional analyses were performed to allow 2-year and 5-year lag in air pollution exposures by including only breast cancer cases diagnosed two years and five years after the exposure measures, respectively, in order to exclude the most recent exposure measures. As no previous studies on air pollution and breast cancer used lagging, our decision was based on minimum lagging period used in other studies of environmental exposures with breast cancer [72] as well as minimum lagging in studies of air pollution and lung cancer [73]. Thus, in the analysis for NO_2_ exposure from 2006, only breast cancer cases diagnosed in 2008 or later were included. Additionally, as our sample included 17,122 women with bilateral oophorectomy (645 of which with breast cancer diagnosis during the follow-up), we conducted a sensitivity analysis that excluded these women. Finally, in the secondary analysis, we excluded 947 women who were diagnosed with ductal carcinoma in situ (DCIS, ICD-9 codes 2330 or ICD-10 code D05).

Prior to regression analysis, we tested the proportional hazards assumption; only age at recruitment violated this assumption. To account for non-proportionality, the interaction term between age at recruitment and time was included in the models [74]. All the tests were two-sided and significance of the effects was assessed at 5% level of significance. All analyses were performed using SAS (SAS Institute Inc. version 9.4).

## Results

In this prospective study of 155,235 postmenopausal women, 6146 developed breast cancer and 149,089 women remained breast cancer-free during the follow-up. The mean age of the study population at enrollment was 60.1 years (range 40–71 years). The average follow-up time calculated for analyses of earliest exposures measured before or at baseline (2005–2006), was 5.4 years (standard deviation [SD] = 3.1 years) for breast cancer cases and 10.7 years (SD = 2.0 years) for cancer-free women. The average follow-up time calculated for analyses of exposures measured in 2010, was 4.9 years (standard deviation [SD] = 2.9 years) for breast cancer cases and 9.8 years (SD = 1.6 years) for cancer-free women. The distribution of air pollution measures (in µg/m^3^) are presented in Table 1 and their correlations are shown in Additional file 1: Table 2. Characteristics of the study population at baseline by breast cancer status are presented in Table 2. As compared to women without breast cancer, women with a breast cancer diagnosis were, on average, older (60.5 vs. 60.1 years, p for difference < 0.0001), had higher BMI (27.6 vs. 27.2 kg/m^3^, p for difference < 0.0001), were more likely to be current postmenopausal hormone therapy users at the time of enrollment (12.2% vs. 9.3%, p for difference < 0.0001), and more likely to have a family history of breast cancer (11.1% vs. 7.2%, p for difference < 0.0001, Table 2). We also present participants’ baseline characteristics by quartiles of the earliest exposure measure (2005 NO_2_) (Additional file 1: Table 3).

**Table 1:** Distribution of air pollution measures in the study sample

Air pollutant (μg/m^3^)	N	Mean	SD	Minimum	25th Percentile	Median	75th Percentile	Maximum
NO_2_ 2005	154,777	29.56	9.93	6.66	22.87	28.04	34.33	126.67
NO_2_ 2006	154,777	28.63	9.03	6.77	22.60	27.60	32.97	129.44
NO_2_ 2007	154,777	30.36	10.54	6.99	23.40	28.58	34.59	138.39
NO_2_ 2010	154,777	26.41	7.45	12.93	21.24	25.95	30.91	107.47
PM_10_ 2007	154,411	22.00	2.86	11.81	20.14	21.72	23.54	36.56
PM_10_ 2010	143,655	16.20	1.89	11.78	15.22	16.01	16.98	30.65
PM_2.5_ 2010	143,655	9.95	1.04	8.17	9.26	9.90	10.52	21.31
PM_2.5 absorbance_ 2010	143,655	1.18	0.27	0.83	0.99	1.12	1.29	4.57
PM _coarse 2.5–10_ 2010	143,655	6.42	0.90	5.57	5.84	6.10	6.63	12.25
NOx 2010	154,777	43.52	15.15	19.74	33.98	41.95	50.17	265.94

NO_2_, Nitrogen dioxide; NOx, Nitrogen oxide; PM_10_, particulate matter ≤ 10 µm in diameter; PM_2.5_, particulate matter ≤ 2.5 µm in diameter; PM_2.5 absorbance_, particulate matter ≤ 2.5 µm in diameter absorbance; PM _coarse 2.5–10_, particulate matter between 2.5 and 10 µm in diameter; SD, Standard deviation

**Table 2:** Characteristics of the study participants at baseline, by breast cancer status

Characteristic	Women who developed breast cancer (N = 6146)	Women without breast cancer (N = 149,089)
*Mean (SD)*		
Age at enrollment, years^a^	60.59 (5.10)	60.06 (5.54)
Follow-up time, years^a^	5.35 (3.14)	10.73 (2.00)
Age at menarche, years^b^	12.93 (1.61)	12.96 (1.59)
Age at menopause, years^a^	50.34 (4.92)	49.72 (5.09)
BMI, kg/m^2a^	27.61 (5.01)	27.15 (5.07)
NO_2_ 2005	29.53 (9.97)	29.57 (9.93)
NO_2_ 2006	28.58 (9.09)	28.64 (9.03)
NO_2_ 2007	30.36 (10.56)	30.37 (10.53)
NO_2_ 2010	26.30 (7.51)	26.41 (7.45)
PM_10_ 2007^b^	22.09 (2.78)	22.00 (2.86)
PM_10_ 2010	16.21 (1.90)	16.20 (1.89)
PM_2.5_ 2010	9.94 (1.04)	9.95 (1.04)
PM_2.5 absorbance_ 2010	1.18 (0.26)	1.18 (0.27)
PM _coarse 2.5–10_ 2010	6.43 (0.91)	6.42 (0.89)
NOx 2010	43.32 (15.22)	43.52 (15.15)
*N (%)*		
Race^b^		
White	5928 (96.45)	142,539 (95.61)
Other	218 (3.55)	6550 (4.39)
Parity/AFB, years^b^		
Nulliparous	1049 (17.07)	24,209 (16.24)
Any children with age at first birth ≤ 25 years	2341 (38.09)	59,787 (40.10)
Any children with age at first birth > 25 years	1904 (30.98)	46,280 (31.04)
Any children with unknown age at first birth	852 (13.86)	18,813 (12.62)
Postmenopausal hormone-replacement therapy^a^		
Never used hormones	2928 (50.12)	74,616 (52.63)
Past	2200 (37.66)	53,915 (38.03)
Current	714 (12.22)	13,239 (9.34)
Family history^a^		
Breast cancer	682 (11.40)	10,776 (7.43)
No breast cancer	5301 (88.60)	134,249 (92.57)
Smoking status^b^		
Never	3490 (56.78)	87,433 (58.65)
Past	2131 (34.67)	49,732 (33.36)
Current	525 (8.54)	11,924 (8.00)
Alcohol intake		
Non-drinker	350 (5.69)	9145 (6.13)
Past drinker	213 (3.47)	5622 (3.77)
Current drinker	5583 (90.84)	134,322 (90.10)

BMI, Body Mass Index; NO_2_, nitrogen dioxide; NOx, Nitrogen oxide; PM_10_, particulate matter ≤ 10 µm in diameter; PM_2.5_, particulate matter ≤ 2.5 µm in diameter; PM_2.5, absorbance_, particulate matter ≤ 2.5 µm in diameter absorbance; PM_coarse 2.5–10_, particulate matter between 2.5 and 10 µm in diameter; SD, standard deviation;

^a^Difference statistically significant at 0.0001 level

^b^Difference statistically significant at 0.05 level

**Table 3:** Associations of particulate matter measures with breast cancer risk (Hazard Ratios [HR] and 95% Confidence Intervals [95% CI])

Year	Air pollution measure	Analyses without exposure lag		Analyses with 2-year exposure lag	
		N with/without breast cancer	HR (95% CI)^a^	N with/without breast cancer	HR (95% CI)^a^
**2007**	**PM**_**10**_				
	per 5 µg/m^3^	6117/148,289	1.09 (1.04, 1.14)	5932/148,055	1.11 (1.06, 1.16)
	per 10 µg/m^3^	6117/148,289	1.18 (1.08, 1.29)	5932/148,055	1.23 (1.12, 1.35)
	Q1: ≤ 20.14 (18.95)	1398/37,298	1.00	1342/37,221	1.00
	Q2: > 20.14 to ≤ 21.72 (20.99)	1605/36,933	1.17 (1.09, 1.26)	1545/36,872	1.18 (1.10, 1.27)
	Q3: > 21.72 to ≤ 23.54 (22.50)	1562/36,972	1.14 (1.06, 1.23)	1512/36,906	1.15 (1.07, 1.24)
	Q4: > 23.54 (25.28)	1552/37,086	1.15 (1.07, 1.24)	1533/37,056	1.19 (1.10, 1.28)
	p for trend	6117/148,289	0.001	5932/148,055	< 0.0001
**2010**	**PM**_**10**_				
	per 5 µg/m^3^	5321/137,115	1.00 (0.93, 1.07)	4281/135,538	0.98 (0.90, 1.06)
	per 10 µg/m^3^	5321/137,115	0.99 (0.86, 1.15)	4281/135,538	0.96 (0.82, 1.12)
	Q1: ≤ 15.22 (14.37)	1363/34,342	1.00	1100/33,942	1.00
	Q2: > 15.22 to ≤ 16.01 (15.68)	1276/34,011	0.95 (0.88, 1.02)	1013/33,597	0.93(0.86, 1.02)
	Q3: > 16.01 to ≤ 16.98 (16.40)	1374/34,512	1.00 (0.93, 1.08)	1118/34,136	1.01 (0.93, 1.10)
	Q4: > 16.98 (18.07)	1308/34,250	0.97 (0.89, 1.04)	1050/33,863	0.96 (0.88, 1.04)
	p for trend	5321/137,115	0.536	4281/135,538	0.524
	**PM**_**2.5**_				
	per 5 µg/m^3^	5321/137,115	0.93 (0.81, 1.06)	4281/135,538	0.91 (0.79, 1.06)
	per 10 µg/m^3^	5321/137,115	0.86 (0.66, 1.12)	4281/135,538	0.84 (0.62, 1.12)
	Q1: ≤ 9.26 (8.76)	1389/34,212	1.00	1122/33,807	1.00
	Q2: > 9.26 to ≤ 9.90 (9.60)	1283/34,191	0.93 (0.86, 1.00)	1031/33,784	0.92 (0.85, 1.01)
	Q3: > 9.90 to ≤ 10.52 (10.18)	1340/34,282	0.97 (0.90, 1.04)	1071/33,898	0.96 (0.88, 1.04)
	Q4: > 10.52 (11.06)	1309/34,430	0.94 (0.87, 1.02)	1057/34,049	0.94 (0.86, 1.02)
	p for trend	5321/137,115	0.217	4281/135,538	0.204
	**PM**_**2.5 absorbance**_				
	per 5 µg/m^3^	5321/137,115	0.90 (0.54, 1.51)	4281/135,538	0.91 (0.51, 1.61)
	per 10 µg/m^3^	5321/137,115	0.82 (0.29, 2.29)	4281/135,538	0.82 (0.26, 2.58)
	Q1: ≤ 0.99 (0.92)	1390/35,218	1.00	1111/34,767	1.00
	Q2: > 0.99 to ≤ 1.12 (1.06)	1306/34,467	0.96 (0.89, 1.04)	1049/34,079	0.97 (0.89, 1.05)
	Q3: > 1.12 to ≤ 1.29 (1.20)	1305/33,233	1.00 (0.93, 1.08)	1051/32,871	1.01 (0.92, 1.09)
	Q4: > 1.29 (1.45)	1320/34,197	0.99 (0.92, 1.07)	1070/33,821	1.00 (0.92, 1.09)
	p for trend	5321/137,115	0.999	4281/135,538	0.835
	**PM**_**coarse 2.5–10**_				
	per 5 µg/m^3^	5321/137,115	1.06 (0.91, 1.23)	4281/135,538	1.02 (0.87, 1.21)
	per 10 µg/m^3^	5321/137,115	1.12 (0.83, 1.51)	4281/135,538	1.05 (0.75, 1.46)
	Q1: ≤ 5.84 (5.71)	1316/33,712	1.00	1041/33,334	1.00
	Q2: > 5.84 to ≤ 6.10 (5.96)	1326/35,038	0.97 (0.90, 1.05)	1091/34,642	1.01 (0.92, 1.10)
	Q3: > 6.10 to ≤ 6.63 (6.30)	1351/33,923	1.02 (0.94, 1.10)	1090/33,512	1.04 (0.95, 1.13)
	Q4: > 6.63 (7.26)	1328/34,442	0.99 (0.92, 1.07)	1059/64,050	1.00 (0.91, 1.08)
	p for trend	5321/137,115	0.984	4281/135,538	0.832
**Cumulative average PM**_**10**_					
	per 5 µg/m^3^	6128/148,640	1.41 (1.32, 1.51)	4903/146,829	1.36 (1.27, 1.47)
	per 10 µg/m^3^	6128/148,640	1.99 (1.75, 2.27)	4903/146,829	1.86 (1.61, 2.15)
	Q1: ≤ 17.92 (17.06)	1398/37,382	1.00	1136/36,993	1.00
	Q2: > 17.92 to ≤ 19.04 (18.52)	1461/37,229	1.05 (0.98, 1.13)	1169/36,808	1.03 (0.95, 1.12)
	Q3: > 19.04 to ≤ 20.25 (19.38)	1461/37,182	1.05 (0.98, 1.13)	1187/36,730	1.05 (0.97, 1.14)
	Q4: > 20.25 (21.39)	1808/36,847	1.35 (1.25, 1.44)	1411/36,298	1.29 (1.19, 1.39)
	p for trend	6128/148,640	< 0.0001	4903/146,829	< 0.0001

NO_2_, nitrogen dioxide; NOx, Nitrogen oxide; PM_10_, particulate matter ≤ 10 µm in diameter; PM_2.5_, particulate matter ≤ 2.5 µm in diameter; PM_2.5 absorbance_, particulate matter ≤ 2.5 µm in diameter absorbance; PM _coarse 2.5–10_, particulate matter between 2.5 and 10 µm in diameterQ1 = 1st quartile; Q2, 2nd quartile; Q3, 3rd quartile; Q4, 4th quartile

^a^Adjusted for age, body mass index, race, age at menopause, age at menarche, parity/age at first birth, postmenopausal hormone use, family history of breast cancer, alcohol consumption, and smoking

### Overall associations of air pollution measures with breast cancer risk

In the main analyses, the risk of breast cancer increased by 18% per 10 µg/m^3^ increase in PM_10_ exposure in 2007 (HR = 1.18, 95%CI 1.08, 1.29, Table 3). Compared to women exposed to the lowest 2007 PM_10_ concentrations, women with higher exposure levels had a greater risk of breast cancer (HR for 4th vs. 1st quartile = 1.15, 95% CI 1.07, 1.24, p-trend = 0.001, Table 3). No association was found for 2010 PM10 exposure. The cumulative average exposure to PM_10_ was significantly positively associated with breast cancer risk, when modeled both as continuous (HR per 10 µg/m^3^ = 1.99, 95% CI 1.75, 2.27), and as quartiles (HR for 4th vs. 1st quartile = 1.35, 95% CI 1.25, 1.44, p-trend < 0.001, Table 3). We found no associations of PM_2.5_, PM_coarse 2.5–10_, PM_2.5 absorbance_, NO_2_, or NOx with breast cancer risk (Tables 3 and 4). The results of associations were similar in sensitivity analysis excluding women with bilateral oophorectomy (data not shown).

**Table 4:** Associations of NO_2_ and NOx with breast cancer risk (Hazard Ratios [HR] and 95% Confidence Intervals [95% CI])

Year	Air pollution measure	Analyses without exposure lag		Analyses with 2-year exposure lag	
		N with/without breast cancer	HR (95% CI)^a^	N with/without breast cancer	HR (95% CI)^a^
**2005**	**NO**_**2**_				
	per 5 µg/m^3^	6130/148,647	1.00 (0.99, 1.02)	6129/148,643	1.00 (0.99, 1.02)
	per 10 µg/m^3^	6130/148,647	1.01 (0.98, 1.03)	6129/148,643	1.01 (0.98, 1.03)
	Q1: ≤ 22.87 (19.46)	1546/37,177	1.00	1546/37,177	1.00
	Q2: > 22.87 to ≤ 28.04 (25.57)	1547/37,145	1.00 (0.93, 1.07)	1547/37,145	1.00 (0.93, 1.07)
	Q3: > 28.04 to ≤ 34.33 (30.79)	1481/37,184	0.96 (0.89, 1.03)	1481/37,184	0.96 (0.89, 1.03)
	Q4: > 34.33 (38.89)	1556/37,141	1.04 (0.97, 1.12)	1556/37,141	1.04 (0.97, 1.12)
	p for trend	6130/148,647	0.390	6129/148,643	0.402
**2006**	**NO**_**2**_				
	per 5 µg/m^3^	6130/148,647	1.00 (0.99, 1.02)	6106/148,624	1.00 (0.99, 1.02)
	per 10 µg/m^3^	6130/148,647	1.00 (0.97, 1.03)	6106/148,624	1.00 (0.97, 1.03)
	Q1: ≤ 22.60 (19.23)	1555/37,173	1.00	1547/37,167	1.00
	Q2: > 22.60 to ≤ 27.60 (25.21)	1567/37,058	1.01 (0.94, 1.08)	1563/37,053	1.01 (0.94, 1.09)
	Q3: > 27.60 to ≤ 32.97 (29.95)	1458/37,251	0.94 (0.87, 1.01)	1452/37,247	0.94 (0.88, 1.01)
	Q4: > 32.97 (38.17)	1550/37,165	1.02 (0.95, 1.09)	1544/37,157	1.02 (0.95, 1.10)
	p for trend	6130/148,647	0.930	6106/148,624	0.905
**2007**	**NO**_**2**_				
	per 5 µg/m^3^	6129/148,643	1.00 (0.99, 1.017)	5944/148,409	1.01 (0.99, 1.02)
	per 10 µg/m^3^	6129/148,643	1.01 (0.98, 1.03)	5944/148,409	1.01 (0.99, 1.04)
	Q1: ≤ 23.40 (19.93)	1548/37,124	1.00	1510/37,062	1.00
	Q2: > 23.40 to ≤ 28.58 (26.16)	1520/37,177	0.98 (0.91, 1.05)	1469/37,110	0.97 (0.90, 1.04)
	Q3: > 28.58 to ≤ 34.59 (31.26)	1496/37,217	0.97 (0.90, 1.04)	1440/37,157	0.95 (0.89, 1.02)
	Q4: > 34.33 (41.77)	1565/37,125	1.04 (0.97, 1.12)	1525/37,080	1.04 (0.97, 1.12)
	p for trend	6129/148,643	0.256	5944/148,409	0.256
**2010**	**NO**_**2**_				
	per 5 µg/m^3^	5552/147,767	0.99 (0.97, 1.01)	4430/146,072	0.99 (0.97, 1.01)
	per 10 µg/m^3^	5552/147,767	0.98 (0.95, 1.02)	4430/146,072	0.98 (0.94, 1.02)
	Q1: ≤ 21.24 (17.90)	1457/36,885	1.00	1165/36,430	1.00
	Q2: > 21.24 to ≤ 25.95 (23.71)	1374/36,995	0.94 (0.88, 1.02)	1090/36,550	0.94 (0.86, 1.02)
	Q3: > 25.95 to ≤ 30.91 (28.35)	1326/36,951	0.91 (0.85, 0.99)	1047/36,650	0.90 (0.83, 0.98)
	Q4: > 30.91 (34.40)	1395/36,936	0.96 (0.89, 1.04)	1128/36,542	0.97 (0.89, 1.05)
	p for trend	5552/147,767	0.190	4430/146,072	0.308
	**NOx**				
	per 5 µg/m^3^	5552/147,767	1.00 (0.99, 1.00)	4430/146,072	0.99 (0.98, 1.00)
	per 10 µg/m^3^	5552/147,767	0.99 (0.97, 1.01)	4430/146,072	0.99 (0.97, 1.01)
	Q1: ≤ 33.98 (27.90)	1430/36,917	1.00	1582/37,127	1.00
	Q2: > 33.98 to ≤ 41.95 (38.23)	1397/36,942	0.98 (0.91, 1.05)	1541/37,148	0.97 (0.89, 1.05)
	Q3: > 41.95 to ≤ 50.17 (45.69)	1394/36,921	0.98 (0.91, 1.05)	1535/37,147	0.97 (0.89, 1.06)
	Q4: > 50.17 (57.73)	1331/36,987	0.93 (0.86, 1.01)	1472/37,225	0.94 (0.86, 1.02)
	p for trend	5552/147,767	0.075	4430/146,072	0.167
**Cumulative average NO**_**2**_					
	per 5 µg/m^3^	6130/148,647	1.00 (0.99, 1.02)	4823/146,718	1.00 (0.99, 1.02)
	per 10 µg/m^3^	6130/148,647	1.00 (0.97, 1.03)	4823/146,718	1.01 (0.97, 1.04)
	Q1: ≤ 22.67 (19.32)	1543/37,164	1.00	1213/36,645	1.00
	Q2: > 22.67 to ≤ 27.64 (25.31)	1557/37,128	1.00 (0.93, 1.08)	1225/36,644	1.01 (0.93, 1.10)
	Q3: > 27.64 to ≤ 33.26 (30.14)	1470/37,230	0.93 (0.86, 1.00)	1135/36,747	0.94 (0.87, 1.02)
	Q4: > 33.26 (38.33)	1560/37,125	1.03 (0.96, 1.11)	1250/36,682	1.03 (0.95, 1.12)
	p for trend	6130/148,647	0.659	4823/146,718	0.683

NO_2_, nitrogen dioxide; NOx, Nitrogen oxide; PM_10_, particulate matter ≤ 10 µm in diameter; PM_2.5_, particulate matter ≤ 2.5 µm in diameter; PM_2.5 absorbance_, particulate matter ≤ 2.5 µm in diameter absorbance; PM _coarse 2.5–10_, particulate matter between 2.5 and 10 µm in diameter Q1, 1st quartile; Q2, 2nd quartile; Q3, 3rd quartile; Q4, 4th quartile

^a^Adjusted for age, body mass index, race, age at menopause, age at menarche, parity/age at first birth, postmenopausal hormone use, family history of breast cancer, alcohol consumption, and smoking

### Lagged exposure analyses

In the analysis with 2-year exposure lag (Tables 3 and 4), we observed a positive association between exposure to 2007 PM_10_ and breast cancer risk (HR per 10 µg/m^3^ = 1.23, 95% CI 1.12, 1.35, Table 3). Compared to women with lowest 2007 PM_10_ exposure, women with higher exposure levels had a greater risk of breast cancer (HR for 4th vs. 1st quartile = 1.19, 95% CI 1.10, 1.28, p-trend < 0.001, Table 3). The cumulative average exposure to PM_10_ was significantly positively associated with breast cancer risk when modeled both as continuous (HR per 10 µg/m^3^ = 1.86, 95% CI 1.61, 2.15), and as quartiles (HR for 4th vs. 1st quartile = 1.29, 95% CI 1.19, 1.39, p-trend < 0.001, Table 3). No significant associations were found for PM_10_ exposure in 2010 or any other air pollutant measures. The associations were similar with 5-year exposure lag (Additional file 1: Table 4).

### Secondary analyses of associations with invasive breast cancer only

In the secondary analyses including 5,175 invasive breast cancer and 148,289 breast cancer-free women, the risk of invasive breast cancer increased by 19% per 10 µg/m^3^ increase in 2007 PM_10_ exposure (HR per 10 µg/m^3^ = 1.19, 95% CI 1.08, 1.31) (Additional file 1: Table 5). Compared to women exposed to lowest concentrations of 2007 PM_10_, women with higher exposure levels had a greater risk of invasive breast cancer (HR for 4th vs. 1st quartile = 1.16, 95% CI 1.07, 1.25, p-trend = 0.002) (Additional file 1: Table 5). The cumulative average exposure to PM_10_ was significantly associated with invasive breast cancer risk when modeled both as continuous (HR per 10 µg/m^3^ = 2.06, 95% CI 1.79, 2.37), and as quartiles (HR for 4th vs. 1st quartile = 1.35, 95% CI 1.25, 1.46, p-trend < 0.001) (Additional file 1: Table 5). We also found a suggestive inverse association between NOx and breast cancer risk (HR for 4th vs. 1st quartile = 0.92, 95% CI 0.84, 0.99, p-trend = 0.035). None of the other air pollution measures were associated with the risk of invasive breast cancer (Additional file 1: Table 5).

In the analysis with 2-year exposure lag, we observed a positive association between 2007 PM_10_ and invasive breast cancer risk (HR per 10 µg/m^3^ = 1.24, 95% CI 1.13, 1.37) (Additional file 1: Table 5). Compared to women with the lowest 2007 PM_10_, women with higher exposure levels had a greater risk of breast cancer (HR for 4th vs. 1st quartile = 1.19, 95% CI 1.10, 1.29, p-trend < 0.001) (Additional file 1: Table 5). The cumulative average exposure to PM_10_ was significantly associated with breast cancer risk, when modeled both as continuous (HR per 10 µg/m^3^ = 1.93, 95% CI 1.65, 2.26), and as quartiles (HR for 4th vs. 1st quartile = 1.30, 95% CI 1.19, 1.42, p-trend < 0.001) (Additional file 1: Table 5). No significant associations were found for PM_10_ exposure in 2010 or any other air pollutant and breast cancer risk (Additional file 1: Table 5).

## Discussion

In this large prospective cohort of postmenopausal women enrolled in the UK Biobank, we investigated the association of air pollution with postmenopausal breast cancer risk. We found positive associations of 2007 PM_10_ and PM_10_ cumulative average with postmenopausal breast cancer risk. In the analysis with a 2-year exposure lag, the associations were stronger for 2007 PM_10_ as compared to overall analysis. No associations were found for other examined air pollution measures.

Our findings for the association of PM_10_ with breast cancer risk are consistent with some, but not all studies [24–28, 30–32, 35]. A recent cohort study from Germany found 19% breast cancer risk increase per 10 μg/m^3^ increase in PM_10_ (Relative Risk [RR] = 1.19, 95% CI 1.09, 1.31) [32]. However, the study population was not limited to postmenopausal women. In an ecological study that utilized region-based national census data that encompassed the entire female population of South Korea for 10 years, Hwang et al. found a 13% breast cancer risk increase per 10 μg/m^3^ increase in PM_10_ level (Odds Ratio [OR] = 1.13, 95% CI 1.09, 1.17) [31]. However, the findings from this ecological study cannot be applied to individuals (ecological fallacy); further, the risk estimates could not be adjusted for breast cancer risk factors. Studies that evaluated the relationship between PM_10_ and breast cancer risk among postmenopausal women have not found any associations. Andersen et al. pooled data from seven European prospective cohorts (overall 3,612 breast cancer cases) and found elevated non-significant associations of PM_10_ with postmenopausal breast cancer (HR per 10 μg/m^3^ = 1.07, 95% CI 0.89, 1.30) [25]. There was considerable heterogeneity between individual cohort estimates, with hazard ratios per 10 μg/m^3^ exposure increase ranging between 0.81 and 1.73. A null association between PM_10_ and postmenopausal breast cancer risk was also observed in the Nurses' Health Study II (HR per 10 μg/m^3^ = 0.97, 95% CI 0.86, 1.09) [28]. However, this cohort is limited to specific female nurses and thus the findings may not be generalizable to other populations. Finally, it is worth to note that there appeared to be a threshold effect rather than a dose–response relationship for associations of PM_10_ with breast cancer risk as the risk estimates for quartiles 2, 3, and 4 were similar.

While we found significant associations of 2007 PM_10_ and cumulative average with postmenopausal breast cancer risk, we did not find an association with 2010 PM_10_, most likely due to PM_10_ concentrations declining over time from a median of 21.72 μg/m^3^ in 2007 to a median of 16.01 μg/m^3^ in 2010 as well as fewer number of breast cancer cases in this analysis. Notably, only 0.07% (n = 95) of all 139,147 women (and 0.17% [n = 6] of breast cancer cases) had a 2010 PM_10_ concentration greater than the 2007 PM_10_ cutoff concentration for the 4th quartile (23.5 μg/m^3^).

We found no associations of PM_2.5_, PM_2.5 absorbance_, or PM_coarse 2.5–10_ with breast cancer risk in any of the analyses performed, consistent with the previous studies in postmenopausal women, though some studies that were not limited to postmenopausal women reported some associations [24, 25, 28, 29]. In the largest cohort study to date including long-term residents of Ontario and registered with Ontario’s provincial health insurance plan on April 1, 1996, the hazard ratio was 1.01 per inter-quartile range (5.3 µg/m^3^) increase of PM_2.5_ (95% CI 1.00, 1.03) [33]. However, the average PM_2.5_ levels in this study were slightly higher than in our sample (10.8 [SD = 3.5] vs. 9.95 [SD = 1.04]) and the analyses were not stratified by menopausal status. Finally, PM_2.5_, PM_2.5_ absorbance and PM coarse _2.5–10_ were only measured in 2010 and not 2007. Therefore, the same reasons for why PM_10_ in 2010 was not associated with breast cancer risk could potentially also explain the null associations for these other PM measures.

To our best knowledge, no other study looked at associations of PM_10_ lagged exposure with postmenopausal breast cancer. We found stronger associations when we included only breast cancer cases diagnosed two years as well as only those diagnosed 5 years after exposure to PM_10_ in 2007; thus, our findings provide further evidence that earlier exposures to higher concentrations of PM_10_ may be more relevant with respect to breast cancer etiology. Even though the confidence intervals for the estimates in overall analysis have some overlap with those in 2-year analysis, this overlap becomes less apparent with 5-year exposure lag further demonstrating that earlier exposures might be more relevant and that the observed differences in the strengths of association is unlikely to be the result of just random variation. However, we found a weaker effect of 2-year or 5-year lagged exposure for PM_10_ cumulative average compared to the effect of exposure for PM_10_ cumulative average without exposure lag, which could be potentially explained by declining PM_10_ concentrations over time combined with a reduction in the number of breast cancer cases included in these lagged analyses. Finally, we could not examine the associations with 7- and 10-year lag in the exposure as this would result in significant reduction of the number of breast cancer cases (by 50–70% of the original Ns, depending on the exposure) for 7-year lagged analysis and down to as few as 70 cases for 10-year lagged analysis), which would not be informative.

Our findings of significant association of PM_10_ with breast cancer risk are consistent with previous studies indicating relevant biological pathways as a possible explanation for potential effects of PM on breast cancer risk which could lead to tumor development. The biological effects of exposure to PMs could result from systemic inflammation, oxidative stress, and epigenetic changes that lead to formation of DNA adducts, disruption of DNA repair, induction of carcinogen-activating enzymes, and DNA methylation of tumor suppressor genes in breast tissue [15–21, 49, 50, 57–59, 75–85]. Importantly, some of these changes are stable and thus low dose long-term exposure would result in accumulation of these alterations over time. Further, some of these compounds have a very long half-life and accumulate in adipose tissue (including the breast) due to their lipophilic properties thus increasing target organ-specific dose [86–88].

Contrary to our hypothesis, we found a marginal association of NOx with breast cancer risk when only invasive cancers were included in analyses. Studies that assessed NO_2_ and NOx exposures reported mixed findings. Six of the 12 studies that investigated the association of NO_2_ with breast cancer risk reported significant positive associations [36, 37]. Of these six studies, two studies investigated the association of NO_2_ stratified by menopausal status, with positive associations found in postmenopausal women [24, 27, 32, 36, 37, 89]*.* Two of the four studies that investigated the association of NOx with breast cancer risk [9, 25, 26, 30] also found significant positive associations [25, 30]. However, in contrast to our study, all the studies except one [25] included premenopausal women and did not stratify by menopausal status. Some of the studies were ecological with no adjustment for known risk factors for breast cancer, or hospital-based case–control studies that included patients diagnosed with bladder cancer among controls, a type of cancer that has been found to be associated with air pollution [30, 32, 33, 36, 90]. As fossil fuel combustion is a major source of NO_2_ and NOx in the air, these two air pollutants may represent a marker of exposure to a mixture of components with carcinogenetic properties, such as PAHs, benzene, metals and other chemicals, possibly acting on breast tissues [23, 61]. Some previous studies had showed that breast cancer risk increases with proximity to roadways and traffic volume [26, 28].

While our findings of suggestive inverse associations of NOx with breast cancer risk are puzzling, they could potentially be the result of residual confounding, for example, by mammographic breast density, a well-established breast cancer risk factor, as some studies reported inverse associations of NOx with high breast density [60]. Information on breast density, however, was not available in UKBiobank. On the other hand, some previous reports have linked long-term exposure to NOx to lower levels of interleukin (IL)-2, IL-8, 40 IL-10 and tumor necrosis factor-α [91], all of which have been implicated in breast carcinogenesis [92]. Importantly, because we observed only association of NOx with invasive breast cancer risk without a clear pattern, future studies are needed to confirm our findings.

Our study is the largest study to date to investigate the association of air pollution with breast cancer among postmenopausal women. The study utilized a population-based prospective cohort with a rigorously defined protocol, rigorous ascertainment of the endpoints through continuous linkages to the national registries, and well-validated methods for assessing air pollution. This study has a few limitations. Information on covariates was used from baseline assessment only; however, the correlations with the values available from follow-up assessments were high (as described in Covariate information section) and thus, use of baseline risk factor data is unlikely to introduce misclassification. We were also unable to investigate associations by breast cancer subtypes, such as estrogen receptor (ER)-positive versus ER-negative, because the information was not recorded within the UK Biobank. Further, exposures to air pollutants were estimated based on a single residential address at baseline; therefore, we cannot rule out potential exposure misclassification caused by outside activities performed away from home or change of residence. However, recent studies suggest very small contribution of commuting to total weekly exposure and demonstrate that omitting commute does not significantly underestimate health effects as compared with the model combining home and work [93, 94]. Any exposure misclassification due to the change in residence is expected to be non-differential and could lead to dilution of the effect. Additionally, the variability in air pollution over time (as demonstrated by the differences in PM_10_ and NO_2_ levels, Table 1) implies that repeated measurements of air pollution recorded over time could provide a better estimate of cumulative exposure. Finally, a large number of potential associations were examined, and some of the significant findings could be false positives as a result of multiple testing.

## Conclusion

We found a significant association of exposure to PM_10_ with postmenopausal breast cancer risk. Our findings contribute to the limited evidence on the association of air pollution with the risk of breast cancer in postmenopausal women. More studies in diverse populations, including Black women and other racial/ethnic minorities, are needed to confirm our results and to elucidate the potential biological mechanisms underlying the observed associations.

## Supplementary Information

**Additional file 1: Table 1.** Summary of the follow-up times and breast cancer case inclusions in different models for associations of air pollution measures with breast cancer risk. **Table 2.** Correlation between 2010 air pollution measures. **Table 3.** Characteristics of the study participants at baseline, by the quartiles of 2005 Nitrogen dioxidelevels. **Table 4.** Associations of 5-year lagged air pollution exposure with breast cancer risk. **Table 5.** Association of air pollution measures with invasive breast cancer risk

## Data Availability

Data used in this study are available from UK Biobank according to standard controlled access procedure. More details can be found at https://www.ukbiobank.ac.uk/enable-your-research/apply-for-access.

## Abbreviations

BMI: Body mass index
CI: Confidence interval
DCIS: Ductal carcinoma in situ
ER: Estrogen receptor
ESCAPE: European Study of Cohorts for Air Pollution Effects
EU: European Union
HR: Hazard ratio
IARC: International Agency for Research on Cancer
ICD: International Classification of Diseases
LUR: Land use regression
MREC: NorthWest Multi-centre Research Ethics Committee
NHS: National Health Service
NOx: Nitrogen oxides
NO_2_: Nitrogen dioxide
OR: Odds ratio
PAHs: Polycyclic aromatic hydrocarbons
PCB: Polychlorinated biphenyls
PCDF: Dibenzofurans
PM: Particulate matter
RR: Relative risk
SD: Standard deviation
UK: United Kingdom
