# Supplementary material for: Association of air pollution with postmenopausal breast cancer risk in UK Biobank
Source: Breast Cancer Res. 2023 Jul 13;25:83. doi: 10.1186/s13058-023-01681-w (PMC10339564; doi:10.1186/s13058-023-01681-w)
Supplement: Supplementary file 1 — Additional file 1: Table 1. Summary of the follow-up times and breast cancer case inclusions in different models for associations of air pollution measures with breast cancer risk. Table 2. Correlation between 2010 air pollution measures. Table 3. Characteristics of the study participants at baseline, by the quartiles of 2005 Nitrogen dioxidelevels. Table 4. Associations of 5-year lagged air pollution exposure with breast cancer risk. Table 5. Association of air pollution measures with invasive breast cancer risk [file 13058_2023_1681_MOESM1_ESM.docx]

**Table 1. Summary of the follow-up times and breast cancer case inclusions in different models for associations of air pollution measures with breast cancer risk**

| **Exposure metric** | **Start of follow-up (year)** | **Incident breast cancers included in the main analyses**  **(years of diagnosis)** | **Incident breast cancers included in the lagged exposure analyses**  **(years of diagnosis)** |
| --- | --- | --- | --- |
| NO_2_ 2005 | 2006 (Recruitment starts) | All cases | Cases only 2007+ |
| NO_2_ 2006 | 2006 (Recruitment starts) | All cases | Cases only 2008+ |
| NO_2_ 2007 | 2007 | Cases only 2007+ | Cases only 2009+ |
| NO_2_ 2010 | 2010 | Cases only 2010+ | Cases only 2012+ |
| Cumulative average NO_2_ | Last available exposure assessment year | All cases | Cases only 2012+ |
| PM_10_ 2007 | 2007 | Cases only 2007+ | Cases only 2009+ |
| PM_10_ 2010 | 2010 | Cases only 2010+ | Cases only 2012+ |
| Cumulative average PM_10_ | Last available exposure assessment year | Cases only 2007+ | Cases only 2009+ |
| PM_2.5_ 2010 | 2010 | Cases only 2010+ | Cases only 2012+ |
| PM_2.5 absorbance_ 2010 | 2010 | Cases only 2010+ | Cases only 2012+ |
| PM _coarse 2.5-10_ 2010 | 2010 | Cases only 2010+ | Cases only 2012+ |
| NOx 2010 | 2010 | Cases only 2010+ | Cases only 2012+ |

**Abbreviations:** NO_2_ - nitrogen dioxide; NOx - Nitrogen oxide; PM_10_ - particulate matter≤10 µm in diameter_;_ PM_2.5_ - particulate matter≤2.5 µm in diameter; PM_2.5 absorbance_ - particulate matter≤2.5 µm in diameter absorbance; PM _coarse 2.5-10_ - particulate matter between 2.5 and 10 µm in diameter;

**Supplementary table 2. Correlation between 2010 air pollution measures ^a^**

| **Air Pollutant** | **NO_2_** | **PM_2.5_** | **PM_10_** | **PM_2.5 absorbance_** | **PM _coarse 2.5-10_** | **NOx** |
| --- | --- | --- | --- | --- | --- | --- |
| **NO_2_** | 1.000 (n=153,319) |  |  |  |  |  |
| **PM_2.5_** | 0.861 (n=142,436) | 1.000 (n=142,436) |  |  |  |  |
| **PM_10_** | 0.506 (n=142,436) | 0.536 (n=142,436) | 1.000 (n=142,436) |  |  |  |
| **PM_2.5 absorbance_** | 0.740 (n=142,436) | 0.599 (n=142,436) | 0.559 (n=142,436) | 1.000 (n=142,436) |  |  |
| **PM _coarse 2.5-10_** | 0.196 (n=142,436) | 0.217 (n=142,436) | 0.814 (n=142,436) | 0.419 (n=142,436) | 1.000 (n=142,436) |  |
| **NOx** | 0.921 (n=154,772) | 0.846 (n=143,650) | 0.513 (n=142,436) | 0.646 (n=142,436) | 0.233 (n=142,436) | 1.000 (n=153,319) |

^a^ All p-values <0.0001

**Supplemental Table 3. Characteristics of the study participants at baseline, by the quartiles of 2005 Nitrogen dioxide (NO_2_) levels**

| **Characteristic** | **Q1: ≤22.87**  **(N=38,723)** | **Q2: <22.87 - ≤28.04 (N=38,692)** | **Q3: <28.04 - ≤34.33 (N=38,665)** | **Q4: >34.33 (N=38,697)** |
| --- | --- | --- | --- | --- |
| ***Mean (SD)*** |  |  |  |  |
| Age at enrollment, years^a^ | 60.25 (5.36) | 60.25 (5.48) | 60.1 (5.56) | 59.72 (5.68) |
| Follow-up time, years^a^ | 11.63 (2.58) | 11.68 (2.60) | 11.64 (2.60) | 11.30 (2.53) |
| Age at menarche, years^b^ | 12.95 (1.57) | 12.94 (1.58) | 12.97 (1.60) | 12.99 (1.63) |
| Age at menopause, years^a^ | 49.97 (5.00) | 49.74 (5.09) | 49.67 (5.17) | 49.62 (5.17) |
| Body Mass Index, kg/m^2a^ | 26.81 (4.81) | 27.23 (4.99) | 27.36 (5.09) | 27.30 (5.35) |
| ***N (%)*** |  |  |  |  |
| Race^a^ |  |  |  |  |
| White | 38,335 (99.00) | 38,052 (98.35) | 37,540 (97.09) | 34,143 (88.23) |
| Other | 388 (1.00) | 640 (1.65) | 1,125 (2.91) | 4,554 (11.77) |
| Parity/AFB, years^a^ |  |  |  |  |
| Nulliparous | 5,793 (14.96) | 5,357 (13.85) | 5,655 (14.63) | 8,285 (21.41) |
| Any children with age at first birth ≤25 yrs | 15,232 (39.33) | 16,504 (42.65) | 16,466 (42.59) | 13,807 (35.68) |
| Any children with age at first birth >25 yrs | 13,155 (33.87) | 12,064 (31.18) | 11,654 (30.14) | 11,263 (29.11) |
| Any children with unknown age at first birth | 4,583 (11.84) | 4,767 (12.32) | 4,890 (12.65) | 5,342 (13.80) |
| Postmenopausal hormone therapy^a^ |  |  |  |  |
| Never used hormones | 18,974 (51.39) | 18,886 (51.38) | 19,032 (51.90) | 20,421 (55.46) |
| Past | 14,495 (39.26 | 14,531 (39.53) | 14,279 (38.94) | 12,651 (34.36) |
| Current | 3,451 (9.35) | 3,344 (9.10) | 3,360 (9.16) | 3,752 (10.19) |
| Family history^a^ |  |  |  |  |
| Breast cancer | 2,961 (7.83) | 2,948 (7.84) | 2,822 (7.50) | 2,695 (7.18) |
| No breast cancer | 34,852 (92.17) | 34,642 (92.16) | 34,814 (92.50) | 34,832 (92.82) |
| Smoking status^a^ |  |  |  |  |
| Never | 23,784 (61.42) | 23,155 (59.84) | 22,488 (58.16) | 21,267 (54.96) |
| Past | 12,598 (32.53) | 12,754 (32.96) | 12,862 (33.27) | 13,473 (34.82) |
| Current | 2,341 (6.05) | 2,783 (7.19) | 3,315 (8.57) | 3,957 (10.23) |
| Alcohol intake^a^ |  |  |  |  |
| Non-drinker | 1,801 (4.65) | 2,098 (5.42) | 2,347 (6.07) | 3,206 (8.28) |
| Past drinker | 1,194 (3.08) | 1,405 (3.63) | 1,511 (3.91) | 1,700 (4.39) |
| Current drinker | 35,728 (92.27) | 35,189 (90.95) | 34,807 (90.02) | 33,791 (87.32) |

**Abbreviations:** Q1 - 1^st^ quartile; Q2 - 2^nd^ quartile; Q3 - 3rd quartile; Q4 - 4^th^ quartile; SD - standard deviation

^a^Difference statistically significant at 0.0001 level

^b^Difference statistically significant at 0.05 level

Supplementary table 4. **Associations of 5-year lagged air pollution exposure with breast cancer risk (Hazard Ratios [HR] and 95% Confidence Intervals [95% CI])**

| **Year** | **Air pollution measure** | **Analyses with 5-year exposure lag** | | | |
| --- | --- | --- | --- | --- | --- |
|  |  | **N with/without breast cancer** | | **HR (95% CI)^a^** | |
| **2005** | **NO_2_** |  | |  | |
|  | per 5 µg/m^3^ | 5,552/147,869 | | 1.01 (0.99, 1.02) | |
|  | per 10 µg/m^3^ | 5,552/147,869 | | 1.02 (0.97, 1.03) | |
|  | Q1: ≤22.87 (19.46) | 1,401/36,968 | | 1.00 | |
|  | Q2: >22.87 - ≤28.04 (25.57) | 1,386/36,926 | | 1.06 (0.92, 1.06) | |
|  | Q3: >28.04 - ≤34.33 (30.79) | 1,312/36,977 | | 1.01 (0.87, 1.01) | |
|  | Q4: >34.33 (38.89) | 1,453/36,998 | | 1.16 (0.99, 1.16) | |
|  | p for trend | 5,552/147,869 | | 0.087 | |
| **2006** | **NO_2_** |  | |  | |
|  | per 5 µg/m^3^ | 4,988/147,049 | | 1.01 (0.99, 1.02) | |
|  | per 10 µg/m^3^ | 4,988/147,049 | | 1.02 (0.97, 1.03) | |
|  | Q1: ≤22.60 (19.23) | 1,256/36,742 | | 1.00 | |
|  | Q2: >22.60 - ≤27.60 (25.21) | 1,270/36,641 | | 1.01 (0.94, 1.10) | |
|  | Q3: >27.60 - ≤32.97 (29.95) | 1,160/36,849 | | 0.93 (0.85, 1.00) | |
|  | Q4: >32.97 (38.17) | 1,302/36,817 | | 1.06 (0.98, 1.45) | |
|  | p for trend | 4,988/147,049 | | 0.317 | |
| **2007** | **NO_2_** |  | |  | |
|  | per 5 µg/m^3^ | 4,430/146,174 | | 1.02 (0.99, 1.02) | |
|  | per 10 µg/m^3^ | 4,430/146,174 | | 1.03 (0.98, 1.03) | |
|  | Q1: ≤23.40 (19.93) | 1,113/36,456 | | 1.00 | |
|  | Q2: >23.40 - ≤28.58 (26.16) | 1,081/36,549 | | 0.96 (0.89, 1.05) | |
|  | Q3: >28.58 - ≤34.59 (31.26) | 1,048/36,578 | | 0.94 (0.86, 1.02) | |
|  | Q4: >34.33 (41.77) | 1,188/36,591 | | 1.10 (0.99, 1.19) | |
|  | p for trend | 4,430/146,174 | | 0.105 | |
|  | **PM_10_** |  | |  | |
|  | per 5 µg/m^3^ | 4,419/145,827 | | 1.18 (1.12, 1.24) | |
|  | per 10 µg/m^3^ | 4,419/145,827 | | 1.39 (1.26, 1.55) | |
|  | Q1: ≤20.14 (18.95) | 950/36,642 | | 1.00 | |
|  | Q2: >20.14 - ≤21.72 (20.99) | 1,160/36,325 | | 1.26 (1.16 1.37) | |
|  | Q3: >21.72 - ≤23.54 (22.50) | 1,128/36,340 | | 1.22 (1.12, 1.33) | |
|  | Q4: >23.54 (25.28) | 1,181/36,520 | | 1.30 (1.20, 1.42) | |
|  | p for trend | 4,419/145,827 | | <0.0001 | |
| **2010** | **NO_2_** |  | |  | |
|  | per 5 µg/m^3^ | 2,692/143,182 | | 0.98 (0.96, 1.00) | |
|  | per 10 µg/m^3^ | 2,692/143,182 | | 0.96 (0.91, 1.01) | |
|  | Q1: ≤21.24 (17.90) | 711/357,44 | | 1.00 | |
|  | Q2: >21.24 - ≤25.95 (23.71) | 654/358,71 | | 0.91 (0.83, 1.02) | |
|  | Q3: >25.95 - ≤30.91 (28.35) | 643/358,09 | | 0.90 (0.81, 1.00) | |
|  | Q4: >30.91 (34.40) | 684/357,58 | | 0.95 (0.85, 1.06) | |
|  | p for trend | 2,692/143,182 | | 0.997 | |
|  | **NOx** |  | |  | |
|  | per 5 µg/m^3^ | 2,692/143,182 | | 0.98 (0.98, 1.00) | |
|  | per 10 µg/m^3^ | 2,692/143,182 | | 0.98 (0.95, 1.00) | |
|  | Q1: ≤33.98 (27.90) | 700/35,772 | | 1.00 | |
|  | Q2: >33.98 - ≤41.95 (38.23) | 668/35,849 | | 0.95 (0.86, 1.06) | |
|  | Q3: >41.95 - ≤50.17 (45.69) | 673/35,764 | | 0.96 (0.86, 1.06) | |
|  | Q4: >50.17 (57.73) | 651/35,797 | | 0.91 (0.82, 1.02) | |
|  | p for trend | 2,692/143,182 | | 0.120 | |
|  | **PM_10_** |  | |  | |
|  | per 5 µg/m^3^ | 2,672/132,901 | | 0.95 (0.86, 1.06) | |
|  | per 10 µg/m^3^ | 2,672/132,901 | | 0.91 (0.74, 1.11) | |
|  | Q1: ≤15.22 (14.37) | 694/33,320 | | 1.00 | |
|  | Q2: >15.22 - ≤16.01 (15.68) | 637/32,918 | | 0.93 (0.83, 1.03) | |
|  | Q3: >16.01 - ≤16.98 (16.40) | 696/33,481 | | 0.99 (0.90, 1.11) | |
|  | Q4: >16.98 (18.07) | 645/33,182 | | 0.93 (0.83, 1.03) | |
|  | p for trend | 2,672/132,901 | | 0.266 | |
|  | **PM_2.5_** |  | |  | |
|  | per 5 µg/m^3^ | 2,672/132,901 | | 0.86 (0.71, 1.03) | |
|  | per 10 µg/m^3^ | 2,672/132,901 | | 0.73 (0.50, 1.07) | |
|  | Q1: ≤9.26 (8.76) | 699/33,181 | | 1.00 | |
|  | Q2: >9.26 - ≤9.90 (9.60) | 637/33,136 | | 0.91 (0.82, 1.02) | |
|  | Q3: >9.90 - ≤ 10.52 (10.18) | 681/33,256 | | 0.97 (0.87, 1.08) | |
|  | Q4: >10.52 (11.06) | 655/33,328 | | 0.92 (0.83, 1.03) | |
|  | p for trend | 2,672/132,901 | | 0.253 | |
|  | **PM_2.5 absorbance_** |  | |  | |
|  | per 5 µg/m^3^ | 2,672/132,901 | | 0.72 (0.34, 1.50) | |
|  | per 10 µg/m^3^ | 2,672/132,901 | | 0.52 (0.12, 2.26) | |
|  | Q1: ≤0.99 (0.92) | 699/34,113 | | 1.00 | |
|  | Q2: >0.99 - ≤1.12 (1.06) | 651/33,438 | | 0.95 (0.86, 1.06) | |
|  | Q3: >1.12 - ≤1.29 (1.20) | 655/32,213 | | 0.99 (0.89, 1.11) | |
|  | Q4: >1.29 (1.45) | 667/33,137 | | 0.98 (0.88, 1.09) | |
|  | p for trend | 2,672/132,901 | | 0.907 | |
|  | **PM _coarse 2.5-10_** |  | |  | |
|  | per 5 µg/m^3^ | 2,672/132,901 | | 1.00 (0.81, 1.23) | |
|  | per 10 µg/m^3^ | 2,672/132,901 | | 1.00 (0.67, 1.53) | |
|  | Q1: ≤5.84 (5.71) | 661/32,675 | | 1.00 | |
|  | Q2: >5.84 - ≤6.10 (5.96) | 666/33,962 | | 0.97 (0.87, 1.08) | |
|  | Q3: >6.10 - ≤6.63 (6.30) | 697/32,901 | | 1.04 (0.94, 1.16) | |
|  | Q4: >6.63 (7.26) | 648/33,363 | | 0.96 (0.87, 1.07) | |
|  | p for trend | 2,672/132,901 | | 0.485 | |
| **Cumulative average NO_2_** | |  | |  | |
|  | per 5 µg/m^3^ | 2,692/143,284 | | 1.00 (0.99, 1.03) | |
|  | per 10 µg/m^3^ | 2,692/143,284 | | 1.02 (0.98, 1.06) | |
|  | Q1: ≤22.67 (19.32) | 669/35,826 | | 1.00 | |
|  | Q2: >22.67 - ≤27.64 (25.31) | 665/35,797 | | 1.00 (0.90, 1.11) | |
|  | Q3: >27.64 - ≤33.26 (30.14) | 627/35,871 | | 0.94 (0.84, 1.05) | |
|  | Q4: >33.26 (38.33) | 731/35,790 | | 1.09 (0.98, 1.21) | |
|  | p for trend | 2,692/143,284 | | 0.176 | |
| **Cumulative average PM_10_** | | |  | |  |
|  | per 5 µg/m^3^ | 2,692/143,282 | | 1.12 (1.01, 1.23) | |
|  | per 10 µg/m^3^ | 2,692/143,282 | | 1.25 (1.02, 1.51) | |
|  | Q1: ≤17.92 (17.06) | 638/36,166 | | 1.00 | |
|  | Q2: >17.92 - ≤19.04 (18.52) | 658/36,036 | | 1.03 (0.93, 1.15) | |
|  | Q3: >19.04 - ≤20.25 (19.38) | 680/35,851 | | 1.07 (0.96, 1.19) | |
|  | Q4: >20.25 (21.39) | 716/35,229 | | 1.14 (1.02, 1.27) | |
|  | p for trend | 2,692/143,282 | | 0.013 | |

**Abbreviations:** NO_2_ - nitrogen dioxide; ; NOx - Nitrogen oxide; PM_10_ - particulate matter≤10 µm in diameter_;_ PM_2.5_ - particulate matter≤2.5 µm in diameter; PM_2.5 absorbance_ - particulate matter≤2.5 µm in diameter absorbance; PM _coarse 2.5-10_ - particulate matter between 2.5 and 10 µm in diameterQ1=1^st^ quartile; Q2 - 2^nd^ quartile; Q3 - 3^rd^ quartile; Q4 - 4^th^ quartile

^a^Adjusted for age, body mass index, race, age at menopause, age at menarche, parity/age at first birth, postmenopausal hormone use, family history of breast cancer, alcohol consumption, and smoking

**Supplemental Table 5. Association of air pollution measures with invasive breast cancer risk (Hazard Ratios [HR] and 95% Confidence Intervals [95% CI])**

| **Year** | **Air pollution measure** | **Analyses without exposure lag** | | | | **Analyses with 2-year exposure lag** | | | |
| --- | --- | --- | --- | --- | --- | --- | --- | --- | --- |
|  |  | **N with/without breast cancer** | | **HR (95% CI)^a^** | | **N with/without breast cancer** | | **HR (95% CI)^a^** | |
| **2005** | **NO_2_** |  | |  | |  | |  | |
|  | per 5 µg/m^3^ | 5,187/148,647 | | 1.01 (0.99, 1.02) | | 5,186/148,643 | | 1.01 (0.992, 1.02) | |
|  | per 10 µg/m^3^ | 5,187/148,647 | | 1.01 (0.98, 1.04) | | 5,186/148,643 | | 1.01 (0.983, 1.04) | |
|  | Q1: ≤22.87 (19.46) | 1,312/37,177 | | 1.00 | | 1,312/37,177 | | 1.00 | |
|  | Q2: >22.87 - ≤28.04 (25..57) | 1,304/37,145 | | 0.99 (0.92, 1.07) | | 1,304/37,145 | | 0.99 (0.92, 1.07) | |
|  | Q3: >28.04 - ≤34.33 (30.79) | 1,256/37,184 | | 0.96 (0.89, 1.04) | | 1,256/37,184 | | 0.96 (0.89, 1.04) | |
|  | Q4: >34.33 (38.89) | 1,315/37,141 | | 1.04 (0.96, 1.12) | | 1,314/37,141 | | 1.04 (0.96, 1.12) | |
|  | p for trend | 5,187/148,647 | | 0.429 | | 5,186/148,643 | | 0.442 | |
| **2006** | **NO_2_** |  | |  | |  | |  | |
|  | per 5 µg/m^3^ | 5,187/148,647 | | 1.00 (1.00, 1.02) | | 5,164/148,624 | | 0.95 (0.87, 1.02) | |
|  | per 10 µg/m^3^ | 5,187/148,647 | | 1.01 (0.98, 1.04) | | 5,164/148,624 | | 1.02 (0.95, 1.11) | |
|  | Q1: ≤22.60 (19.23) | 1,311/37,173 | | 1.00 | | 1,303/37,167 | | 1.00 | |
|  | Q2: >22.60 - ≤27.60 (25.21) | 1,333/37,058 | | 1.02 (0.944, 1.01) | | 1,329/37,053 | | 1.02 (0.94, 1.09) | |
|  | Q3: >27.60 - ≤32.97 (29.95) | 1,235/37,251 | | 0.94 (0.87, 1.02) | | 1,229/37,247 | | 0.94 (0.88, 1.01) | |
|  | Q4: >32.97 (38.17) | 1,308/37,165 | | 1.02 (0.95, 1.10) | | 1,303/37,157 | | 1.02 (0.95, 1.10) | |
|  | p for trend | 5,187/148,647 | | 0.914 | | 5,164/148,624 | | 0.881 | |
| **2007** | **NO_2_** |  | |  | |  | |  | |
|  | per 5 µg/m^3^ | 5,186/148,643 | | 1.01 (0.99, 1.02) | | 5,021/148,409 | | 1.01 (0.994, 1.02) | |
|  | per 10 µg/m^3^ | 5,186/148,643 | | 1.01 (0.99, 1.04) | | 5,021/148,409 | | 1.02 (0.988, 1.04) | |
|  | Q1: ≤23.40 (19.93) | 1,313/37,124 | | 1.00 | | 1,280/37,062 | | 1.00 | |
|  | Q2: >23.40 - ≤28.58 (26.16) | 1,280/37,177 | | 0.97 (0.90, 1.05) | | 1,234/37,110 | | 0.96 (0.89, 1.04) | |
|  | Q3: >28.58 - ≤34.59 (31.26) | 1,264/37,217 | | 0.96 (0.89, 1.04) | | 1,214/37,157 | | 0.95 (0.88, 1.03) | |
|  | Q4: >34.33 (41.77) | 1,329/37,125 | | 1.04 (0.96, 1.13) | | 1,293/37,080 | | 1.04 (0.96, 1.13) | |
|  | p for trend | 5,186/148,643 | | 0.241 | | 5,021/148,409 | | 0.248 | |
|  | **PM_10_** |  | |  | |  | |  | |
|  | per 5 µg/m^3^ | 5,175/148,289 | | 1.09 (1.04, 1.15) | | 5,010/148,055 | | 1.11 (1.06, 1.17) | |
|  | per 10 µg/m^3^ | 5,175/148,289 | | 1.19 (1.08, 1.31) | | 5,010/148,055 | | 1.24 (1.13, 1.37) | |
|  | Q1: ≤20.14 (18.95) | 1,183/37,298 | | 1.00 | | 1,137/37,221 | | 1.00 | |
|  | Q2: >20.14 - ≤21.72 (20.99) | 1,349/36,933 | | 1.17 (1.08, 1.26 | | 1,293/36,872 | | 1.16 (1.08, 1.26) | |
|  | Q3: >21.72 - ≤23.54 (22.50) | 1,329/36,972 | | 1.15 (1.06, 1.24) | | 1,284/36,906 | | 1.15 (1.07, 1.25) | |
|  | Q4: >23.54 (25.28) | 1,314/37,086 | | 1.16 (1.07, 1.25) | | 1,296/37,056 | | 1.19 (1.10, 1.29) | |
|  | p for trend | 5,175/148,289 | | 0.002 | | 5,010/148,055 | | 0.0001 | |
| **2010** | **NO_2_** |  | |  | |  | |  | |
|  | per 5 µg/m^3^ | 4,685/147,767 | | 0.99 (0.97, 1.01) | | 3,737/146,072 | | 0.99 (0.97, 1.01) | |
|  | per 10 µg/m^3^ | 4,685/147,767 | | 0.98 (0.95, 1.02) | | 3,737/146,072 | | 0.98 (0.94, 1.03) | |
|  | Q1: ≤21.24 (17.90) | 1,233/36,885 | | 1.00 | | 977/36,430 | | 1.00 | |
|  | Q2: >21.24 - ≤25.95 (23.71) | 1,156/36,995 | | 0.94 (0.87, 1.02) | | 927/36,550 | | 0.95 (0.87, 1.04) | |
|  | Q3: >25.95 - ≤30.91 (28.35) | 1,131/36,951 | | 0.92 (0.85, 1.00) | | 893/36,650 | | 0.92 (0.94, 1.00) | |
|  | Q4: >30.91 (34.40) | 1,165/36,936 | | 0.95 (0.88, 1.03) | | 940/36,542 | | 0.96 (0.88, 1.05) | |
|  | p for trend | 4,685/147,767 | | 0.190 | | 3,737/146,072 | | 0.295 | |
|  | **NOx** |  | |  | |  | |  | |
|  | per 5 µg/m^3^ | 4,685/147,767 | | 1.00 (0.99, 1.00) | | 3,737/146,072 | | 0.99 (0.98, 1.01) | |
|  | per 10 µg/m^3^ | 4,685/147,767 | | 0.99 (0.97, 1.01) | | 3,737/146,072 | | 0.99 (0.97, 1.01) | |
|  | Q1: ≤33.98 (27.90) | 1,215/36,917 | | 1.00 | | 962/37,127 | | 1.00 | |
|  | Q2: >33.98 - ≤41.95 (38.23) | 1,187/36,942 | | 0.98 (0.90, 1.06) | | 940/37,148 | | 0.98 (0.89, 1.07) | |
|  | Q3: >41.95 - ≤50.17 (45.69) | 1,174/36,921 | | 0.97 (0.89, 1.05) | | 943/37,147 | | 0.98 (0.89, 1.07) | |
|  | Q4: >50.17 (57.73) | 1,109/36,987 | | 0.92 (0.84, 0.99) | | 892/37,225 | | 0.92 (0.84, 1.01) | |
|  | p for trend | 4,685/147,767 | | 0.035 | | 3,737/146,072 | | 0.100 | |
|  | **PM_10_** |  | |  | |  | |  | |
|  | per 5 µg/m^3^ | 4,486/137,115 | | 1.00 (0.93, 1.08) | | 3,609/135,538 | | 0.99 (0.91, 1.08) | |
|  | per 10 µg/m^3^ | 4,486/137,115 | | 1.01 (0.86, 1.18) | | 3,609/135,538 | | 0.98 (0.82, 1.16) | |
|  | Q1: ≤15.22 (14.37) | 1,156/34,342 | | 1.00 | | 933/33,942 | | 1.00 | |
|  | Q2: >15.22 - ≤16.01 (15.68) | 1,084/34,011 | | 0.95 (0.87, 1.03) | | 851/33,597 | | 0.92 (0.84, 1.01) | |
|  | Q3: >16.01 - ≤16.98 (16.40) | 1,128/34,512 | | 0.97 (0.90, 1.06) | | 925/34,136 | | 0.99 (0.90, 1.08) | |
|  | Q4: >16.98 (18.07) | 1,118/34,250 | | 0.97 (0.90, 1.06) | | 900/33,863 | | 0.97 (0.88, 1.06) | |
|  | p for trend | 4,486/137,115 | | 0.618 | | 3,609/135,538 | | 0.695 | |
|  | **PM_2.5_** |  | |  | |  | |  | |
|  | per 5 µg/m^3^ | 4,486/137,115 | | 0.92 (0.80, 1.07) | | 3,609/135,538 | | 0.92 (0.76, 1.08) | |
|  | per 10 µg/m^3^ | 4,486/137,115 | | 0.85 (0.64, 1.14) | | 3,609/135,538 | | 0.85 (0.62, 1.17) | |
|  | Q1: ≤9.26 (8.76) | 1,183/34,212 | | 1.00 | | 950/33,807 | | 1.00 | |
|  | Q2: >9.26 - ≤9.90 (9.60) | 1,082/34,191 | | 0.92 (0.85, 1.00) | | 868/33,784 | | 0.92 (0.84, 1.01) | |
|  | Q3: >9.90 - ≤ 10.52 (10.18) | 1,120/34,282 | | 0.95 (0.88, 1.03) | | 898/33,898 | | 0.95 (0.86, 1.04) | |
|  | Q4: >10.52 (11.06) | 1,101/34,430 | | 0.93 (0.86, 1.01) | | 893/34,049 | | 0.94 (0.85, 1.03) | |
|  | p for trend | 4,486/137,115 | | 0.145 | | 3,609/135,538 | | 0.225 | |
|  | **PM_2.5 absorbance_** |  | |  | |  | |  | |
|  | per 5 µg/m^3^ | 4,486/137,115 | | 0.95 (0.54, 1.67) | | 3,609/135,538 | | 1.02 (0.55, 1.89) | |
|  | per 10 µg/m^3^ | 4,486/137,115 | | 0.91 (0.30, 2.77) | | 3,609/135,538 | | 1.03 (0.30, 3.58) | |
|  | Q1: ≤0.99 (0.92) | 1,176/35,218 | | 1.00 | | 934/34,767 | | 1.00 | |
|  | Q2: >0.99 - ≤1.12 (1.06) | 1,107/34,467 | | 0.97 (0.89, 1.05) | | 893/34,079 | | 0.98 (0.89, 1.07) | |
|  | Q3: >1.12 - ≤1.29 (1.20) | 1,086/33,233 | | 0.98 (0.91, 1.07) | | 875/32,871 | | 1.00 (0.91, 1.09) | |
|  | Q4: >1.29 (1.45) | 1,117/34,197 | | 0.99 (0.91, 1.08) | | 907/33,821 | | 1.01 (0.92, 1.11) | |
|  | p for trend | 4,486/137,115 | | 0.981 | | 3,609/135,538 | | 0.781 | |
|  | **PM _coarse 2.5-10_** |  | |  | |  | |  | |
|  | per 5 µg/m^3^ | 4,486/137,115 | | 1.08 (0.92, 1.27) | | 3,609/135,538 | | 1.04 (0.87, 1.25) | |
|  | per 10 µg/m^3^ | 4,486/137,115 | | 1.16 (0.84, 1.60) | | 3,609/135,538 | | 1.09 (0.76, 1.57) | |
|  | Q1: ≤5.84 (5.71) | 1,119/33,712 | | 1.00 | | 878/33,334 | | 1.00 | |
|  | Q2: >5.84 - ≤6.10 (5.96) | 1,127/35,038 | | 0.97 (0.89, 1.05) | | 92934,642 | | 1.02 (0.93, 1.11) | |
|  | Q3: >6.10 - ≤6.63 (6.30) | 1,112/33,923 | | 0.99 (0.91, 1.07) | | 899/33,512 | | 1.01 (0.92, 1.11) | |
|  | Q4: >6.63 (7.26) | 1,128/34,442 | | 0.99 (0.91, 1.07) | | 903/64,050 | | 1.01 (0.92, 1.11) | |
|  | p for trend | 4,486/137,115 | | 0.984 | | 3,609/135,538 | | 0.999 | |
| **Cumulative average NO_2_** | | |  | |  | |  | |  |
|  | per 5 µg/m^3^ | 5,187/148,647 | | 1.00 (0.99, 1.02) | | 4,074/146,718 | | 1.01 (0.99, 1.03) | |
|  | per 10 µg/m^3^ | 5,187/148,647 | | 1.01 (0.98, 1.05) | | 4,074/146,718 | | 1.01 (0.98, 1.05) | |
|  | Q1: ≤22.67 (19.32) | 1,302/37,164 | | 1.00 | | 1,019/36,645 | | 1.00 | |
|  | Q2: >22.67 - ≤27.64 (25.31) | 1,323/37,128 | | 1.00 (0.93, 1.09) | | 1,042/36,644 | | 1.02 (0.94, 1.12) | |
|  | Q3: >27.64 - ≤33.26 (30.14) | 1,245/37,230 | | 0.94 (0.87, 1.02) | | 952/36,747 | | 0.94 (0.86, 1.02) | |
|  | Q4: >33.26 (38.33) | 1,317/37,125 | | 1.04 (0.95, 1.12) | | 1,061/36,682 | | 1.05 (0.96, 1.14) | |
|  | p for trend | 5,187/148,647 | | 0.610 | | 4,074/146,718 | | 0.584 | |
| **Cumulative average PM_10_** | | |  | |  | |  | |  |
|  | per 5 µg/m^3^ | 5,185/148,640 | | 1.43 (1.34, 1.54) | | 4,143/146,829 | | 1.39 (1.28, 1.50) | |
|  | per 10 µg/m^3^ | 5,185/148,640 | | 2.06 (1.79, 2.37) | | 4,143/146,829 | | 1.93 (1.65, 2.26) | |
|  | Q1: ≤17.92 (17.06) | 1,185/37,382 | | 1.00 | | 957/36,993 | | 1.00 | |
|  | Q2: >17.92 - ≤19.04 (18.52) | 1,224/37,229 | | 1.04 (0.96, 1.12) | | 978/36,808 | | 1.03 (0.94, 1.12) | |
|  | Q3: >19.04 - ≤20.25 (19.38) | 1,239/37,182 | | 1.05 (0.97, 1.14) | | 1,008/36,730 | | 1.06 (0.97, 1.16) | |
|  | Q4: >20.25 (21.39) | 1,537/36,847 | | 1.35 (1.25, 1.46) | | 1,200/36,298 | | 1.30 (1.19, 1.42) | |
|  | p for trend | 5,185/148,640 | | <.0001 | | 4,143/146,829 | | <.0001 | |

**Abbreviations:** NO_2_ - nitrogen dioxide; PM_10_ - particulate matter≤10 µm in diameter_;_ PM_2.5_ - particulate matter≤2.5 µm in diameter; PM_2.5 absorbance_ - particulate matter≤2.5 µm in diameter absorbance; PM _coarse 2.5-10_ - particulate matter between 2.5 and 10 µm in diameter; NOx - Nitrogen oxide; Q1 - 1^st^ quartile; Q2 - 2^nd^ quartile; Q3 - 3rd quartile; Q4 - 4^th^ quartile;

^a^Adjusted for age, body mass index, race, age at menopause, age at menarche, parity/age at first birth, postmenopausal hormone use, family history of breast cancer, alcohol consumption, and smoking
